# Questioning the Mpemba effect: hot water does not cool more quickly than cold

**DOI:** 10.1038/srep37665

**Published:** 2016-11-24

**Authors:** Henry C. Burridge, Paul F. Linden

**Affiliations:** 1 Department of Applied Mathematics and Theoretical Physics, University of Cambridge, Centre for Mathematical Sciences, Wilberforce Road, Cambridge CB3 0WA, UK; 2 Department of Civil and Environmental Engineering, Imperial College London, Skempton Building, South Kensington Campus, London SW7 2AZ, UK

## Abstract

The Mpemba effect is the name given to the assertion that it is quicker to cool water to a given temperature when the initial temperature is higher. This assertion seems counter-intuitive and yet references to the effect go back at least to the writings of Aristotle. Indeed, at first thought one might consider the effect to breach fundamental thermodynamic laws, but we show that this is not the case. We go on to examine the available evidence for the Mpemba effect and carry out our own experiments by cooling water in carefully controlled conditions. We conclude, somewhat sadly, that there is no evidence to support meaningful observations of the Mpemba effect.

The statement “hot water does not cool more quickly than cold” is vague and imprecise; hot water can be made to cool more quickly than cold by supplying more energy to the cooling of hot water, but it is under such a non-specific premise that the Mpemba effect has become an artefact in popular science. More precisely, we show that for two samples of water, identical except for a difference in initial temperature, cooled under the same conditions to a prescribed temperature (for example, the freezing temperature of water) the initially hotter sample will take longer to cool — contrary to the assertion of the Mpemba effect. Despite the non-specific nature of the effect, the Mpemba effect has been the subject of numerous articles in international broadsheet newspapers (e.g. refs 1, 2, 3) and was the focus of a competition organised in 2012 by the Royal Society of Chemistry (RSC) which received substantial publicity, for example a special report on the BBC’s Newsnight programme. As such the Mpemba effect cannot simply be disregarded. Moreover, both The Telegraph^2^ and The Daily Mail^4^ have reported that the scientific work of a group of chemists working in Singapore^5^ provides a molecular mechanism to explain the effect. These findings, and those of ref. 6, concern the molecular interactions and hydrogen bonding within liquid water. While the findings of these studies are of great interest in their own right, we note that small-scale molecular effects are parameterised within the thermal and fluid properties of water — properties which are known to a reasonable degree of accuracy and do indeed vary with temperature. Such findings therefore offer a route to explaining the Mpemba effect only if they result in a meaningful hysteresis in the thermal or fluid properties of water. A model exhibiting a hysteresis in the cooling of water is presented by ref. 5 which is compared to an observation of the Mpemba effect documented as part of a popular science competition organised by the RSC^7^ — we include the experimental observations by ref. 7 within the data analysed in the present study. It is our aim to present an investigation of the history behind the Mpemba effect, examine the scientific evidence for it, consider the underlying physical mechanisms for the effect and determine whether the effect actually exists in any meaningful manner.

There is no clear universally accepted scientific definition of the ‘Mpemba effect’. Mpemba & Osborne^8^ document the time for freezing to commence while others include the freezing process (for example see ref. 9). This lack of clarity is reflected by the level of discrepancies in the literature, which offers a number of different explanations. Broadly speaking, when two samples of water are cooled to the same temperature, in the same manner with the two samples being identical except for their initial temperature, and the initially hotter sample cools in less time, one can consider the Mpemba effect to have been observed. The temperature at which cooling times are compared has often been chosen to be 0 °C (or below) making careful measurements more difficult because of the phase change that occurs as water freezes.

Observations of hot water freezing in less time than cold water date back to classical science. Aristotle^10^ noted that the ancient Greeks of Pontus exploited the effects when they encamped on the ice to fish, and similar observations have been repeated by Bacon^11^ and Descartes^12^. More modern awareness of this apparent anomaly range from the accidental experiments of the Tanzanian school boy, Mpemba (after whom the phenomenon is popularly known^8^), to the competition calling for explanations of the phenomenon by the RSC.

The Mpemba effect is an oft cited scientific anomaly and has been widely used in high-school and undergraduate physics projects^13^,^14^. The effect may appear anomalous since on first consideration one might regard the first law of thermodynamics to be breached. An interpretation of the first law is that the change in the internal energy of a closed system is equal to the amount of heat supplied (accounting for any work done on or by the system). Thus, in the absence of work, for a constant heat flux one naturally expects hot water to take longer to cool to freezing than cooler water. However, typically the cooling does not occur in environments which can be regarded as inducing a constant heat flux, instead most cooling occurs in (near) constant temperature environments. One example of this being the widespread domestic formation of ice-cubes within ice-trays, for which the ice-trays typically sit on a cold plate within a freezer and are cooled by the thermostatically controlled freezer which acts to maintain an approximately constant temperature. Hence, an ice-tray filled with warm water experiences a larger temperature difference and, therefore, a larger initial heat flux compared with an ice-tray filled with cooler water. Moreover, in the presence of an initially hot sample the freezer may remain on, and doing work, to drive the cooling for longer. This, however, by no means explains the Mpemba effect — the hot water must take some time to cool to the initial temperature of the cooler sample of water, after which *all else being equal* one would expect the further cooling of the warm sample to take the same time as the cooling of the colder sample. Hence the warm water, in total, would take longer to cool. Thus for the Mpemba effect to be observed there must be some difference in the chemistry of the samples or the physics of their cooling either initially or when at equivalent temperatures — understanding and examining the various mechanisms that might give rise to such differences remains the focus of scientific debate.

The winning entry to the RSC competition, for example, cites four factors as possibly contributing to the Mpemba effect, namely: (a) evaporation, (b) dissolved gases, (c) mixing by convective currents, and (d) supercooling^7^. No doubt all four processes affect the cooling rate of water, albeit to differing extents, and crucially their effects may be strongly coupled. For example, in two volumes of water, only differing in initial temperature and then cooled in identical conditions, one would expect that different convective currents might develop. Therefore, for significantly different initial temperatures the characteristic times that a given water particle remains in contact with an imperfection in the container or impurity within the water (e.g. dissolved gases) would vary between the two samples and so the level of supercooling required to form ice crystals would vary also. Thus it can be reasoned that the observed variations in the extent to which supercooling occurs must arise, at least in part, due to differences in convective currents and the relative levels of dissolved gases (further affected if evaporation occurs). Hence all the factors which have been proposed to individually cause the Mpemba effect may alter the extent of supercooling required to cause water to freeze.

A reasonable start to analysing the problem is to consider the process in two stages; first, cooling the water to an average temperature of 0 °C (or enthalpy equivalent thereof), and second, freezing the water to form solid ice. In so doing any effects associated with the supercooling of water are entirely contained within the second stage. We restrict our definition of the Mpemba effect to the first stage of the process, i.e. the process of cooling a sample of warm water to 0 °C in less time than it takes to cool a sample of water, which is notionally identical except that it is initially at a lower temperature, to 0 °C.

## Three widely cited historical references to Mpemba-like effects in water

The cooling and freezing of water has intrigued some great scientific minds. Aristotle, Sir Francis Bacon and René Descartes have all been credited with consideration of the Mpemba effect^15^ and, although this list is by no means comprehensive, it is worth documenting the precise observations of these three renowned scientists.

In his treatise on earth sciences (therein “Meteorology”) Aristotle^10^, book I part 12 is concerned with the freezing of water and contains the following text: *The fact that the water has previously been warmed contributes to its freezing quickly: for so it cools sooner. Hence many people, when they want to cool hot water quickly, begin by putting it in the sun. So the inhabitants of Pontus when they encamp on the ice to fish* (*they cut a hole in the ice and then fish*) *pour warm water round their reeds that it may freeze the quicker, for they use the ice like lead to fix the reeds.*

The reference to ice as ‘like lead’ in connection to fishing potentially raises confusion since the use of lead to weight fishing lines is widespread in traditional fishing; ice being less dense than water clearly makes it unsuitable for weighting fishing lines. It is our interpretation of the description ‘like lead to fix the reeds’ that it refers to the stiffening of the reeds by the formation of ice so that the reeds can be plunged beneath the water, hence avoiding the need to weight the reeds so that they might sink. It would, therefore, seem that Aristotle and the peoples of ancient Greece believed that warming water did make it freeze faster.

The second book (section L in ref. 11) of Sir Francis Bacon’s “Aphorisms on the interpretation of nature or on the kingdom of man”, includes a lengthy discourse regarding “Mans works on natural bodies” including a discussion of heat and cold; within which, while concerned with matters of medicine, he states: *We should also deal with the preparation of substances to receive cold: for example, slightly warm water will freeze more easily than water which is altogether cold, and so on.*

No further discussion nor details are provided. Indeed, it is not clear whether any of Bacon’s experiments actually concerned the freezing of water^16^ and hence the observations leading to, or the source of, his stated belief that warm water cools faster than cold is also unclear.

In his essay on Meteorology, near the end of his first discourse, René Descartes^12^ describes some experiments in which both hot and cold water are frozen within a beaker and states: *we can also see by experiment that water which has been kept hot for a long time freezes faster than any other sort, because those of its parts which can least cease to bend evaporate while it is being heated.*

The ‘bending’ which he describes, refers to his hypothesis for the motion of particles and although he credits evaporation for hot water freezing faster, his description of the experimental beaker indicates it has a long thin neck (which aided his observations of the expansion and contraction of water as it was heated and cooled) but would have restricted the area of the free-surface and hence evaporation. It is, therefore, unlikely that evaporation was the dominant physical effect leading to Descartes’ observation. However, like Aristotle and Bacon, Descartes does seem to document observations or convictions that can be fairly described as indicating that they would have supported assertions that the ‘Mpemba effect’ is genuine.

## More recent scientific investigations of the Mpemba effect

The, now popular, adoption of the name ‘Mpemba effect’ is owed to the lack of freezer space at a Tanzanian school. While making ice-cream one pupil placed his mixture of milk and sugar in the freezer without first boiling it; another pupil, Mpemba, worried that he would not find space in the freezer and put his boiling mixture straight into the freezer without first allowing it to cool. Both pupils returned an hour and a half later to find Mpemba’s mixture had frozen while the other had not^8^. Mpemba did not brush this curious observation aside, instead he asked friends (some of whom made a living selling ice-cream and apparently exploited the time saving effects of this anomalous behaviour) and teachers to explain his observations but to no avail. Mpemba eventually asked a visiting lecturer from the University of Dar es Salaam to explain his observations. The open-minded Dr Osborne was intrigued by Mpemba’s observation and later began investigating the effect with his students, ultimately publishing a scientific paper with Mpemba on the observed effect^8^. The ‘Mpemba effect’ also appears to be widely accepted in the Northern Americas^17^. In the same month that Mpemba & Osborne^8^ published their findings a chemist working in Canada^18^ published an article on the very same subject. In his article, Kell describes centuries-old Canadian ‘folklore’ of wooden pails being left out to freeze, and the pails containing the hot water freezing fastest.

Certain subsequent studies report being unable to observe the effect, for example, Ahtee^19^ who examined the fraction of ice formed and Hsu^9^ who considered the time taken for the samples to form solid ice. However, other studies report being able to reproduce the effect, typically, using domestic style ice formation. Numerous differences exist between the experimental conditions of these various studies. These variations include: altering the nature of the cooling supplied, e.g. insulating the base^8^,^20^, submerging samples in cooling baths (for example^21^,^22^) and radiative cooling^23^; degassing or deionising the samples (for example see refs 14 and 24); the addition of dissolved gases^25^ and controlling or monitoring evaporation from the sample (see ref. 23). Despite this wealth of experimental data, detailed analysis is typically lacking; for example, almost all studies present the absolute sample temperature rather than the sample temperature relative to the cooling environment and typically no consideration is given to the volume (mass) of water being cooled nor the geometry of the cooling vessel. A notable exception is the study of Maciejewski^17^ who analysed his data in terms of nondimensional parameters, the Grashof (*Gr*), Prandtl (*Pr*) and Rayleigh (*Ra*) numbers, concluding that the key parameter is *GrPr*^3^ and that the Mpemba effect may be driven by convection.

A number of studies have proposed physical models for the freezing of water in connection with the Mpemba effect. Katz^26^ developed a freezing front model based on a ‘Stefan problem’ with a moving boundary condition which is unable to predict the effect, while the models of Kell^18^, Vynnycky and co-workers^27^,^28^ consider the effects of evaporative cooling, suggesting that evaporation alone is sufficient to observe the Mpemba effect. Vynnycky and co-workers include an experimental observation of the Mpemba effect based on temperature measurements near the water surface. However, they also note that different cooling curves were obtained for samples with identical initial temperatures and that they had difficulty in repeatedly reproducing any observations of the Mpemba effect, citing uncontrollable “micro-physical processes” as the cause of such variations. Vynnycky and Kimura^29^ present results from a detailed experimental examination, and a theoretical model, for the cooling of water in the context of the Mpemba effect. Their experimental results reporting the time at which solidification begins, show no evidence to support the Mpemba effect. However, their data reporting results for the time at which the layer of ice had grown to a particular thickness (therein 25 mm) “hinted at a freezing time inversion, and hence the Mpemba effect”. They attribute such effects to supercooling and they go on to suggest that their experimental data indicates that supercooling is more likely to occur with lower initial temperatures — a suggestion that would promote Mpemba-like effects in water.

Recent advances in the understanding of the bonding of water molecules have been suggested as a potential route to explaining the Mpemba effect which requires a hysteresis within the molecular interactions dependent on the initial temperature. A model accounting for the relaxation dynamics of the hydrogen bonds in liquid water has been proposed^5^ in which a ‘cell’ of water is considered to comprise of the ‘bulk’ (90% of its volume) and the ‘skin’ (10% of its volume). For selected values of the ratio of thermal diffusivity between the skin and bulk (approximately a 50% difference in diffusivity), termed ‘skin supersolidity’, the model exhibits cooling akin to the Mpemba effect. The results of the model are qualitatively compared to an experimental observation of the Mpemba effect documented as part of the 2012 competition organised by the RSC^7^. Through an experimental investigation of the behaviour during cooling of tetrahydrofuran hydrate (a clathrate hydrate)^30^, it is reported that the “formation kinetics of [tetrahydrofuran] hydrate therefore might depend on its initial temperature” and suggest this is Mpemba-like behaviour. The advances in the understanding of the molecular interactions within water, and clathrate hydrates, may be of some significance in understanding the Mpemba effect. However, for this to be the case it would require that the bulk thermal and/or fluid properties of water are significantly influenced by the initial, i.e. the history of the, temperature of the water — it is not yet clear that this is the case. Should it be shown to be necessary it would, indeed, be a result of real significance; for example, standard reference tables for the properties of water would need to be updated to account for not only the current temperature but also the route to the said temperature.

## Results

### Analysis of our ‘Mpemba style’ data and the data from other studies

**Figure 1:**
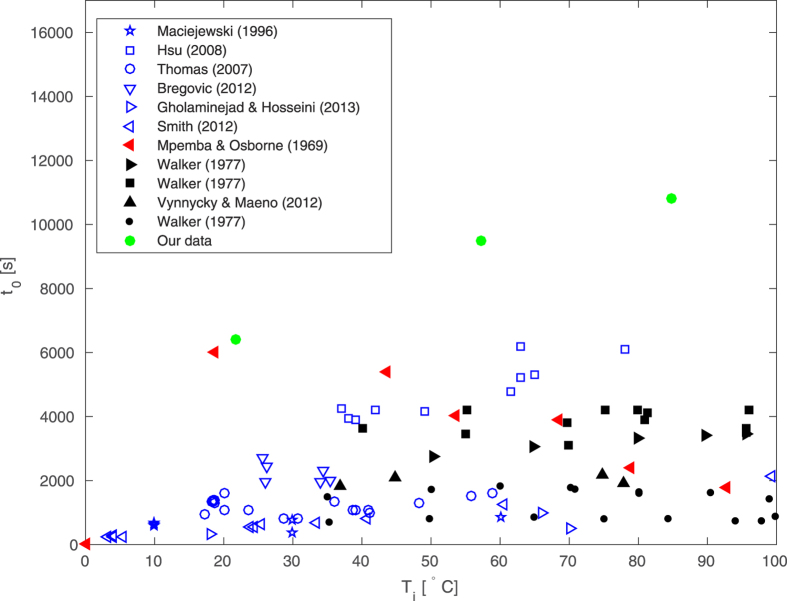
The time *t*_0_ to cool to 0 °C, plotted against the initial temperature, *T*_*i*_ for the ‘Mpemba-type’ experiments. The data show a broad trend of increasing cooling time with increasing initial temperature, with the notable exception being the data of Mpemba & Osborne^8^.

Figure 1 plots the variation in the time *t*_0_, to cool samples to 0 °C, with the initial temperature from a variety of studies including our ‘Mpemba-type’ experiments. We have attempted to represent a broad selection of published experimental data regarding the Mpemba effect. We note that the data from the careful experiments of 29 reporting the time to cool to 0 °C (their Fig. 5), which exhibited no evidence of the Mpemba effect, could not be included due to difficulties in accurately obtaining data from their printed figure. Their results for the time to for the ice layer to grow to a depth of 25 mm cannot be fairly included in our analysis, since we exclude the freezing process; however, we discuss these results when drawing our conclusions. The mass of water, the geometry of its container and indeed the nature of the cooling varied widely between the different datasets and this variation is reflected in the spread of the data. From Fig. 1 it is difficult to draw any conclusions from the data, except that broadly speaking the cooling time increases with initial temperature. The only exception, which reports data (across a broad range of temperatures) that exhibit a decreasing trend in cooling time with increasing initial temperature, is that of Mpemba & Osborne^8^.

**Figure 2:**
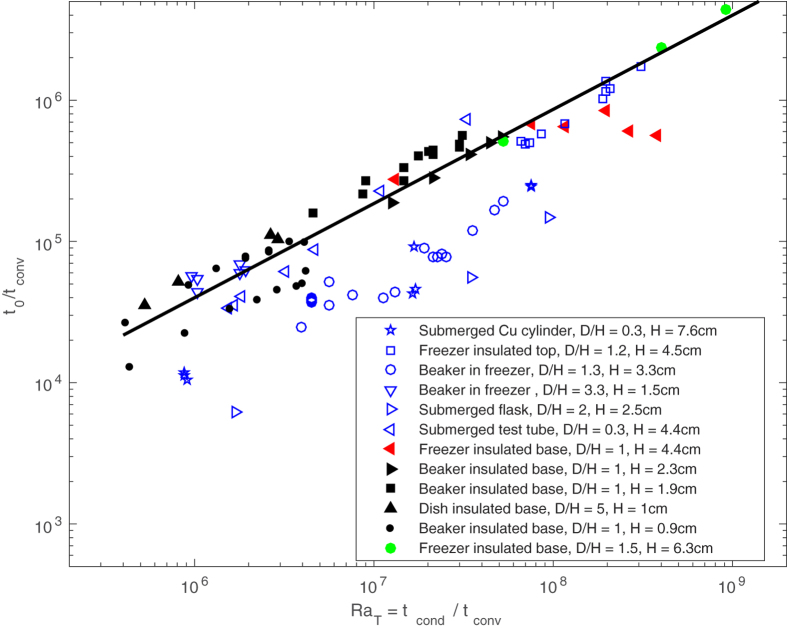
The data from Fig. 1 scaled to show variation of *t*_0_/*t*_*conv*_ (the time to cool to 0 °C in units of the convective time scale) with Rayleigh number, *Ra*_*T*_ = *t*_*cond*_/*t*_*conv*_. The ‘stably cooled’ data are marked by blue open symbols and ‘convectively dominated’ data are marked by solid symbols. The black solid line marks the scaling for high-Rayleigh number convective cooling, (5).

Figure 2 shows the variation in the cooling time *t*_0_, scaled by the convective time scale, with the temperature averaged Rayleigh number from the various studies detailed in Fig. 1 (for details of the convective time scale and the temperature averaged Rayleigh number see the Methods section). Some of the studies included in Fig. 2 did not explicitly provide all the details required to scale the data, and in such cases we made reasonable estimates based on the information provided (details of which are also provided in our Methods section). The experimental conditions vary widely between the eight independent studies from which data are included within the figure. There is no obvious systematic bias for the cooling times based on the geometry of the cooling vessel, despite the aspect ratio of width to height, *D/H*, varying by a factor of fifteen and the depth of water being cooled varying by a factor of eight within the data — indicating that the geometry may be appropriately reflected by the length scales within the temperature averaged Rayleigh number *Ra*_T_. There is, however, an obvious bias in the cooling times based on the nature of the cooling and we broadly split the data into two datasets. The first set we describe as ‘convectively dominated’ data (marked by the solid symbols in Fig. 2) which broadly consists of samples where the base was insulated or cooling from below was inhibited in some manner (see the legend in Fig. 2 for details). In such cases there is no direct heat transfer between the freezer base (or cooling plate) and the sample of water is predominately cooled through the sides or top of the sample and unstable density stratifications are promoted. In such cases, the heat transfer is inhibited by the addition of insulation and hence the cooling times are typically increased, despite the increased role of convection. The second dataset we describe as ‘stably cooled’ (marked by the blue hollow symbols in Fig. 2) which consists of data for which the heat flux through the base of the sample is expected to have been significant (e.g. where the sample was placed directly on a cooling plate), and the cooling is expect to have promoted stably stratified sample of water (at least above 4 °C).

The data within each individual dataset exhibit a broadly consistent trend, with the cooling time increasing with *Ra*_T_ and the datasets are best-fit (in a least squares sense) by a power law of approximately 
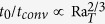
. This suggests that the cooling times follow


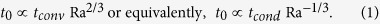


We note that we scaled the data in Fig. 1 using a number of alternative definitions for the Rayleigh number, for example taking all parameters at the initial conditions or combining individually temperature-averaged parameters to form the Rayleigh number, *cf.* Equation (7). The different definitions of the Rayleigh number that we tested all resulted in the various datasets exhibiting trends well approximated by (1).

Considerations of high Rayleigh number convection, in which the assumption that the heat flux is independent of the depth of the fluid, imply that


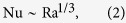


(for example, see ref. 31) where Nu = *Q*/(*κ*Δ*T/H*) is the Nusselt number, with *κ* the thermal diffusivity of the fluid, *Q* being proportional to the flux of heat and Δ*T* being a characteristic temperature difference between the fluid and the cooled surface. The time rate of change of temperature for a given sample is then proportional to the heat flux, i.e. *Q*, and given that *Ra* ∼ *β*Δ*TgH*^3^/(*κv*), from equation (2) we can write


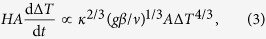


where *β* and *v* are the coefficient of thermal expansion and the kinematic viscosity of the fluid, and *A* is the cooled surface area of the fluid. Hence


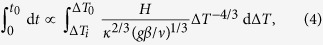


where 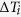 and 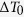 are the initial and final characteristic temperature differences (between the fluid and the cooled surface). Thus


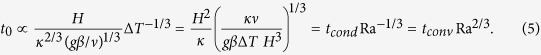


We note that crucially, in deriving (5) we assumed that the convection exhibited behaviour associated with that of asymptotically high Rayleigh number convection. The data investigating the Mpemba effect, plotted in Fig. 2 (obtained at initial Rayleigh numbers up to *O*(10^10^)), fits well with the trend predicted by (5) suggesting that the experimental data can be regarded as high Rayleigh number. As such, if the data plotted in Fig. 2 are shown not to exhibit the Mpemba effect, as indeed we go on to argue, then one must expect that data obtained at higher Rayleigh numbers would also not exhibit the Mpemba effect.

### Analysis of the occurrence of the Mpemba effect

The above analysis, although informative as to the physics of cooling water, does not explicitly address when the Mpemba effect has been observed. In order to establish a single observation of the Mpemba effect, one must compare two experiments which are identical in every manner except for a difference in the initial temperatures of the water samples. One can then state that the Mpemba effect may be regarded to have been observed if the sample of water initially at the higher temperature reaches the desired cooling temperature first. To illustrate when the Mpemba effect may be reported to have been observed we consider the average rate at which heat is transferred *Q* from the initially hot *Q*_*H*_ and initially cold *Q_C_* samples, where for a given sample *Q* = Δ*E/t*_*0*_ = (*E*_*i*_ − *E*_0_)/*t*_*0*_ ∝ Δ*T/t*_0_ = (*T*_*i*_ − *T*_0_)/*t*_0_ with *E*_*i*_ and *E*_0_ denoting the initial and final enthalpy of the samples, respectively.

**Figure 3:**
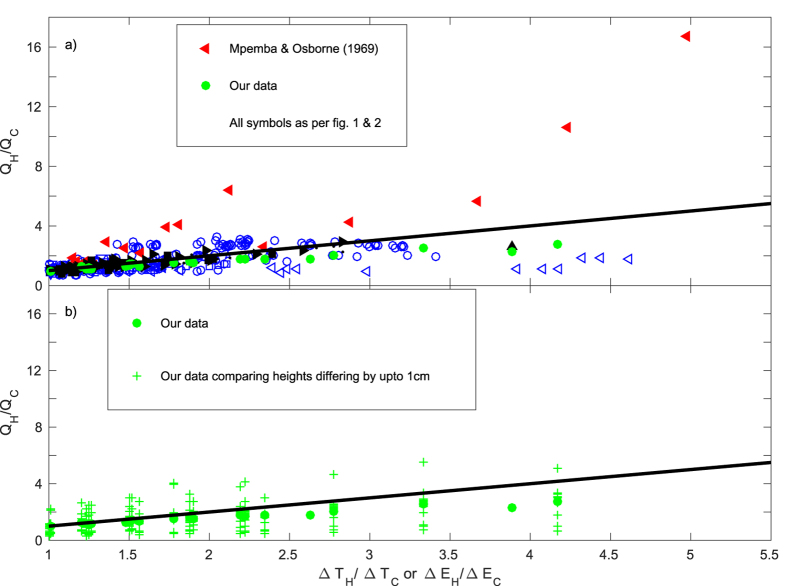
The variation in the ratio of mean heat transfer rates with initial temperature (or equivalently enthalpy) for pairs of otherwise identical samples of hot and cold water. (**a**) The historical data shown in Fig. 1 and a summary of our ‘second-type’ experiments. (**b**) The results of our ‘second-type’ experiments. The black solid lines mark *Q*_H_/*Q*_*C*_ = Δ*T*_*H*_/Δ*T*_*C*_. The green crosses (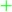) in (**b**) show the data we would report if the height at which we measure the temperature was inaccurate by 1 cm.

The Mpemba effect can be reported as having been observed when the inequality *Q*_*H*_/*Q_C_* > Δ*E*_H_/Δ*E_C_* is satisfied, since *Q*_*H*_/*Q_C_* > Δ*E*_H_/Δ*E_C_* ⇒ *t*_*c*_ > *t*_*H*_, where *t*_*c*_ and *t*_*H*_ denote the cooling time of the cold and hot samples, respectively. Figure 3(a) plots the variation in the ratio *Q*_*H*_/*Q_C_* with Δ*E*_H_/Δ*E_C_* (or equivalently Δ*T*_H_/Δ*T_C_*) for the various pairs of data shown in Fig. 1 and the results of our experiments of the ‘second-type’ (see the Methods section). Figure 3(b) highlights the results of our experiments of the ‘second-type’, with an allowance for spatial variation in the temperature measurements. The relationship *Q*_*H*_/*Q_C_* = Δ*E*_H_/Δ*E_C_* is marked by solid black lines within Fig. 3. Hence, any data lying above this line may be reasonably reported as an observation of the Mpemba effect.

Examining Fig. 3a shows that the majority of the data reported lie below the ‘Mpemba effect line’ (*Q*_*H*_/*Q_C_* = Δ*E*_H_/Δ*E_C_*) and hence the Mpemba effect was clearly not observed in these cases. Data from a number of studies do lie on or just above Mpemba effect line. Notably, these data tend to be towards the left hand end of the horizontal axis, i.e. the temperature of the hotter sample is only marginally greater than that of the cooler sample. This suggests that any inaccuracies in the measurement of temperature may be significant. There are two datasets which are exceptions to this finding, namely, Mpemba & Osborne^8^ and Thomas^14^. None of the data of Thomas^14^ lie far above the Mpemba effect line. Indeed, Fig. 3b plots our data from our ‘second-type’ experiments, i.e. those designed to avoid any formation of ice, in which we recorded the temperatures at a range of different heights within each sample. In addition to our data deduced by comparing temperatures recorded at equal heights within the hotter and cooler samples, Fig. 3b includes the data (marked 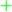) which we would have reported if the vertical positions at which we recorded the temperature were incorrectly measured by up to 1 cm. These data show observations which lie above the Mpemba effect line and as such could, quite incorrectly, be described as being observations of the Mpemba effect if sufficient care had not been taken in our experiments. The vertical and horizontal location of this data within the figure encompasses the region that includes all the data reporting to be observations of the Mpemba effect in other studies. Hence, if in any particular set of experiments the vertical position of the temperature measurements were incorrect, by just 1 cm, then from the data of those experiments one could (again, quite incorrectly) conclude that the Mpemba had been observed. We note that in studies reporting observations of the Mpemba effect the authors are either unable to produce the effect in a repeatable manner or details pertaining to the precise height of the temperature measurements were not reported. The only study which includes observations beyond the region covered by our data shown in Fig. 3b is that of Mpemba & Osborne^8^, which includes observation that lie both far above the Mpemba effect line and also towards the right-hand end of the horizontal axis — we note that these data show significant scatter from any physically reasonable trend.

We have made efforts to contact both of the authors, Mr Erasto B. Mpemba and Dr Denis Osborne. In our attempts to contact Dr Osborne we were saddened to be informed of his death in September 2014. It seems that throughout his life, Dr Osborne continued to make extremely positive contributions to both science and politics. We have so far failed in our attempt to contact Mr Mpemba although we understand he was the principal game officer in the Tanzanian Ministry of Natural Resources and Tourism, Wildlife Division (he is now retired). We have been unable to deduce the source of any systematic error in the experimental procedure or experimental set-up of Mpemba & Osborne^8^ that could feasibly have led to such extreme data being recorded.

### Discussion and Conclusions

We conclude that despite our best efforts, we were not able to make observations of any physical effects which could reasonably be described as the Mpemba effect. Moreover, we have shown that all data (with the only exceptions coming from a single study) reporting to be observations of the Mpemba effect within existing studies fall just above the Mpemba effect line, i.e. the difference in the cooling times between the hot and cold samples is marginal. We have shown (Fig. 3) that much of the data reporting to be observations of the Mpemba effect were from studies not reporting the height at which temperatures were measured^7^,^14^,^20^,^21^,^22^,^23^ and that the conclusions drawn from these data could have been altered by simply recording temperatures without precisely monitoring the height. Indeed, all the data which lie just above the Mpemba effect line in Fig. 3 (including data for which the temperautre measurement height was carefully monitored and reported^17^,^24^,^28^) are, by the very nature of experiments, subject to some degree of uncertainty which may ultimately affect whether the observed results are recorded as an apparent observation of the Mpemba effect or not. To be precise regarding our meaning by this statement, let us now consider the reported observations of the Mpemba effect from, arguably, the two most careful sets of experiments within the literature^28^,^29^. The study^28^ does present data for one observation of the Mpemba effect but also reports obtaining “different cooling curves even if the initial temperatures were identical”, furthermore they state “[c]areful and precise experiments to probe the Mpemba effect can be tried by cooling hot and cool water in two similar containers simultaneously, but it is extremely difficult to obtain scientifically meaningful and reproducible results”. The study^29^ shows a potential observation of the Mpemba effect (in the times for the ice layer to grow to a thickness of 25 mm, their figure 19) for a single pair of initial temperatures (from a possible 21 initial temperature pairings), namely the pair of initial temperatures 10 °C and 15 °C. From data recorded at a fixed height (for example, 5 mm) the samples cooling from 15 °C exhibit a mean cooling time of approximately 95 minutes while those cooling from 10 °C the mean is approximately 105 minutes — hence in taking only the mean of the data for this particular temperature pairing one could describe the Mpemba effect as having been observed. However, the variation in notionally identical experiments is significant. At the same recording height, for samples cooling from 15 °C the recorded time spans the range 95–105 minutes while for samples cooling from 10 °C the recorded time spans the range 100–110 minutes. As such, the variation in notionally identical experiments is at least large enough to render any conclusion that the Mpemba effect has been observed in the mean data as highly questionable, and so this cannot be regarded as a meaningful observation of the effect.

The only exception to our above statements, the single study in which some data is reported that shows dramatically warmer samples cooling in substantially less time (i.e. data points that are far above the line *Q*_*H*_/*Qc* = Δ*T*_H_/Δ*Tc* in Fig. 3) is the data reported by Mpemba & Osborne^8^. If these data could be reproduced in a repeatable fashion and the underlying mechanism understood then it would be of real significance to a multitude of applications relying on the transfer of heat. For example ref. 8, report cooling a sample from 90 °C to freezing point in 30 minutes while a sample at 20 °C took 100 minutes to cool to freezing point, i.e. the average heat transfer rate during cooling was observed to increase by a factor of 15 by simply increasing the initial temperature of the sample. With the use of modern heat-exchangers such a result would have profound implications for the efficiency of any number of common industrial processes. However, over the subsequent 47 years, numerous studies have attempted to demonstrate the ‘effect’ on a scale comparable to that reported by Mpemba & Osborne. Despite these efforts, including our own, none have succeeded. We must therefore assert that this particular dataset may be fundamentally flawed and thus, unless it can be shown to be reproducible and repeatable, this dataset must be regarded as erroneous.

We must highlight that our primary focus has been to examine the cooling of water to the freezing point (observed under standard atmospheric conditions), i.e. an enthalpy equivalent of 0 °C. In so doing we have been able to show that much of the published experimental data exhibit a scaling behaviour associated with asymptotically high Rayleigh number convection. Thus one cannot expect to observe samples of hot water cooling to 0 °C faster than colder samples by carrying out experiments at higher Rayleigh numbers. Under our definition of the Mpemba effect, akin to the definition in the ‘original’ paper by Mpemba & Osborne^8^ (in which they documented “the time for water to start freezing”) we are forced to conclude that the ‘Mpemba effect’ is not a genuine physical effect and is a scientific fallacy.

If one extends the definition of the Mpemba effect to include the freezing process then one can examine the experimental evidence presented by a number of scientific studies which have sought to include the effect of freezing, e.g. refs 9,21,22,28 and 29. The freezing of water to ice is a thermodynamically intensive process. For example, the energy required to change the phase of a given mass of water at 0 °C, into ice at 0 °C is approximately equal to the energy required to cool the same mass of water from 80 °C to 0 °C in the liquid state. Intuition, therefore, guides one to expect the time to completely freeze a sample of water could depend only weakly on the initial water temperature. Moreover, freezing is initiated by a nucleation process and as such it is susceptible to variations at the smallest physical scales, e.g. imperfections in the surface of containers or impurities within the water samples — the physical scales of which are extremely difficult to control in even the most precise experiments. Such intuition is entirely born out in the experimental evidence, with no single study able to report repeatable observations of the Mpemba effect when the freezing process is included^9^,^21^,^22^,^28^,^29^. Experimental observations of a particular example of warm water cooling and freezing in less time than a particular example of initially cooler water have been made — what is yet to be reported is any experimental evidence that samples of water can be consistently cooled and frozen in less time (the time being less by a repeatable and statistically significant amount) by simply initiating the cooling from a higher temperature. As such we can conclude that even with the freezing process included within the definition of the Mpemba effect, the Mpemba effect is not observable in any meaningful way.

We are not gladdened by such a conclusion, indeed quite the opposite. The Mpemba effect has proved to be a wonderful puzzle with which to engage and interest people of all ages and backgrounds in the pursuit of scientific understanding. However, the role of scientists is to objectively examine facts and further knowledge by reporting the conclusions, and as such we feel compelled to disseminate our findings. Finally, we want to give hope to the educators who may have previously relied on the Mpemba effect as a useful tool with which to inspire their students. There are numerous genuine artefacts of science which can continue to provide such inspiration. For example, try filling two identical glasses, one with fresh water and one with salty water (both of equal temperature), place a few cubes of ice in each and observe which melts first — many students will be surprised by the result, finding it counter to their experience and intuition. Equally one could try placing a thin sheet of card on top of a glass of water, turn the glass upside down and then remove your hand from the card — watch as the atmospheric air pressure allows the water to be held in the glass — repeat this, replacing the card by just a rigid gauze with holes of up to a few millimetres and still the water will be held within the glass^32^. We hope that these examples serve to act as catalysts for those seeking other examples of genuine science and that these help to inspire scientific interest within future generations.

## Methods

### Dimensional considerations

The physics of cooling water within a regular three dimensional vessel, all surfaces of which are held at a uniform temperature, can be described in the terms of a thermal buoyancy potential *g *′, three length scales *L*_*x*_, *L*_*y*_, *L*_*Z*_, and the kinematic viscosity *v* and thermal diffusivity *κ* for water. It is common in both the practical cooling of water (e.g. the domestic formation of ice-cubes) and the experiments reported in the literature that the two horizontal length scales are of similar magnitude, and herein we assume *L*_*x*_ ≈ *L*_*y*_ = *D* (where *D* is a characteristic width or diameter of the cooling vessel) and denote the vertical length scale *L*_*z*_ = *H*, where *H* is the depth of water being cooled. As such, the problem can be described by three non-dimensional variables and it is appropriate to select the Grashof number, G*r* = *g *′*H*^3^/*v*^2^ (cf. the Reynolds number for inertial flows); Prandtl number, P*r* = *v/κ*; and the aspect ratio *D/H*. These three non-dimensional parameters can all be combined within a Rayleigh number for the cooling.

Within a fluid heat may be transported either by advection (convection) or thermal diffusion (conduction); the Rayleigh number can be interpreted as a ratio of the time scales for conduction, *t*_*cond*_, and convection, *t*_*conv*_. Suitable length scales for the Rayleigh number can be identified by consideration of these time scales. Conduction, or thermal diffusion, acts to distribute heat in all directions and so *t*_*cond*_ ∝ L^2^/*κ* ∝ min(*H*^2^, *D*^2^)/*κ*, as conduction will predominantly occur over the shortest length scale of the cooling vessel (since this must be the direction of the strongest temperature gradients). Convection is generated when thermal effects give rise to gravitationally unstable distributions of density and so it is appropriate to consider only the vertical length scale *H* in the convective time scale. Hence an appropriate Rayleigh number for the cooling is *Ra* = *g *′*H*^3^ min(1, *D/H*)^2^/(*κv*) = G*r* × P*r* × min(1, *D/H*)^2^.

A suitable representation for the thermal buoyancy potential *g*′ is worthy of consideration. It is natural to define the buoyancy as the gravitational acceleration scaled by the normalised density difference between two relevant fluids. One might argue that it is appropriate to take the difference between the density of water at the initial temperature and at some other temperature, e.g. 0 °C (see the definition of the Grashof number in ref. 17); however, so doing highlights two particular issues. First, the buoyancy can only ever be an indicative scale of the driving cooling potential since one would not expect that, within a given sample, water still at the initial temperature would directly interact with water at 0 °C. Second, such a definition does not account for the differences in the cooling times that one would expect if the same sample were placed in a cooling environment held at 0 °C or in an environment at a far lower temperature, e.g. −50 °C. Consequently, it is more appropriate to accept *g *′ as an indicative scale for the driving cooling potential and, as such, define the thermal buoyancy potential by


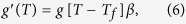


where *T*_*f*_ is the temperature of the cooling environment, *T* is the characteristic instantaneous temperature of the water being cooled, and *β* = *β(T*) is the coefficient of thermal expansion for water at the temperature *T*. Given the density maximum of water at about 4 °C, over the relevant temperature range, 0 °C ≤ *T* ≤ 100 °C, the coefficient of thermal expansion and hence the buoyancy will change sign if a given sample cools below 4 °C. Furthermore, both the kinematic viscosity and thermal diffusivity of water vary with temperature, in the case of the viscosity by factor of six over the temperature range of cooling^33^. In order to account for varying physical properties of water as it cools we consider a temperature averaged Rayleigh number, which incorporates values of *β(T*) calculated from the variations in the density of water with temperature from^34^, values of *κ(T*) calculated from the density^34^, thermal conductivity^35^ and specific heat capacity^36^ of water, and *v(T*) taken from^33^.

We define the temperature averaged Rayleigh number *Ra*_*T*_, for water cooling from an initial temperature *T*_*i*_ to a final temperature *T*_0_, as


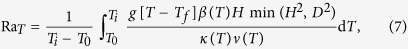


the time scale for conduction as


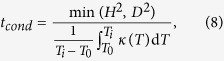


and maintaining the Rayleigh number as the ratio of times scales for conduction and convection gives the time scale for convection as


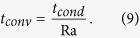


### Experiments

We carried out two types of experiments: the first was designed to mimic the experiments of Mpemba & Osborne^8^, and the second was designed to avoid any formation of ice, and thereby avoid issues associated with phase change, by keeping the cooling plate at 0.3 °C. For both sets of experiments temperatures were digitally recorded and stored using up to eight thermocouples, with a data-logger connected to a computer running LabVIEW. The thermocouples were calibrated using a refrigerated circulator providing temperatures accurate to within 0.01 °C.

In the first set of experiments, our ‘Mpemba style’ experiments, three samples of water each of mass 400 g (measured to an accuracy of within 0.1%) were placed within glass beakers of approximate diameter *D* = 9.0 cm; filling the beaker with a water depth of approximately *H* = 6.3 cm. All the samples of water were boiled, to remove some of the dissolved gases, and then left to cool for varying amounts of time so that the three samples were at different initial temperatures *T*_*i*_ = {21.8, 57.3, 84.7} °C, respectively. The samples were then placed on a 5 cm thick sheet of expanded polystyrene sitting inside a standard domestic chest-freezer. All three samples were placed inside the freezer at the same time in order to ensure that the samples were exposed to the same cooling from the thermostatically controlled chest-freezer. The thermostat on the freezer was set to −18 °C. On placing the samples into the freezer the ambient air temperature within was observed to rise but, after approximately 15 minutes, the freezer temperature had cooled back down to −18 °C. Subsequently, the freezer temperature gradually increased (due to the imperfect insulation of the freezer) until it reached approximately −15 °C at which point the thermostat activated the freezer refrigeration unit and the freezer temperature was cooled once again to −18 °C. This periodic cooling and warming of the freezer, in the temperature range −18 °C ≤ *T*_*f*_ ≤ −15 °C, continued throughout the experiment. Prior to being placed inside the freezer a thermocouple was located and carefully fixed centrally within each sample of water. The temperature of the thermocouples within each water samples were recorded at 1 second intervals throughout the experiment and the time taken for the temperature of each sample to first fall to 0 °C denoted as *t*_0_ = {6397, 9504, 10812}s, respectively.

In the second set of experiments we filled a perspex tank, of horizontal cross-section 20 cm × 20 cm, with fresh water to a depth of 10 cm. Expanded polystyrene sheets (5 cm thick) were attached to the base and the four sides of the tank to act as insulation. The water was then cooled by carefully suspending a brass cooling plate such that the cooling plate was in direct contact with the upper surface of the water. The cooling plate had been carefully machined so that it contained a continuous channel, entirely housed within the plate except for openings at two of its corners which were connected to insulated pipes. The channel meandered within the plate so that by passing ethylene glycol solutions (continuously cooled by a Thermo Haake refrigerated circulator, Phoenix-line, model PII-C41P) through the channel the entire plate was held at an approximately uniform and constant temperature. The refrigerated circulator included a reservoir containing 15 000 cm^3^ of ethylene glycol solution cooled by a refrigeration cycle of power of approximately 1 kW. The circulator passed the solution through insulated pipes and around the machined channel (of cross-section less than 1 cm^2^) at approximately 400 cm^3^/s.

In these experiments of ‘the second type’, to avoid the formation of ice the cooling plate was held at a temperature of 0.3 °C. Prior to our experiments, seven T-type thermocouples (Omega, HSTC-TT-TI-24 S-5 M) were carefully positioned and clamped in place at specified heights within the tank. The thermocouples had been calibrated, to an accuracy of 0.01 °C using the refrigerated circulator, over a temperature range of −20 °C and 100 °C. Throughout each experiment, temperatures were recorded from each of the thermocouples at a frequency of 1 Hz using a National Instruments 9213 measurement system and digitally stored in csv files for later analysis within Matlab. The characteristic temperature of the water at any instant was determined by spatially averaging the temperatures recorded at the thermocouples positioned at the carefully measured heights. Experiments were run until the water within the tank reached a steady temperature which took approximately one day to occur. Since in these experiments the temperature of the samples were intended to remain above freezing point, we defined the cooling time based on the time taken to cool to 4 °C (this temperature being selected to maximise the role of convection), the times to cool to this temperature were in the range 12–17 hrs. It is important to note that since our experiments of ‘the second type’ were deliberately never cooled to 0 °C data from these experiments cannot be included in Figs 1 or 2, and is only included in Fig. 3 in which only the relative cooling times of hot and cold samples are compared. As such, our results are not affected by the choice (in our experiments of the second-type) to measure the time to cool to 4 °C — identical trends in our data are observed with any reasonable variation in this choice of the target temperature. During these experiments the initial temperature *T*_*i*_ of the fresh water was systematically varied between experiments in the range 18 °C ≤ *T*_*i*_ ≤ 75 °C.

### Assumptions made in sourcing the data of other studies

In order to be able to scale the data published in other studies it was necessary to have sufficient information in order to be able to calculate the Rayleigh number, i.e. *T*_*i*_, *T*_*f*_, *H* and *D*, see equation (7). For certain studies^9^,^17^,^20^,^28^ the required information was explicitly provided. Table 1 provides details of information not explicitly provided by the remaining studies for which we report data. In each case, details of our assumptions and the data on which these assumptions was based is provided. It should be noted that the sensitivity of our results to the assumptions detailed in the table is by no means dramatic. Indeed, any reasonable variations to our assumptions does not alter any of our findings.

## Additional Information

**How to cite this article**: Burridge, H. C. and Linden, P. F. Questioning the Mpemba effect: hot water does not cool more quickly than cold. *Sci. Rep.* **6**, 37665; doi: 10.1038/srep37665 (2016).

**Publisher’s note:** Springer Nature remains neutral with regard to jurisdictional claims in published maps and institutional affiliations.

## Figures and Tables

**Table 1:** Assumptions made in order to include the data published in other studies and the underlying evidence provided by each of those studies.

Study	Information provided	Assumed information
Mpemba^8^	‘Icebox of a domestic refrigerator’	*T*_*f*_ = −15 °C
Thomas^14^	50 ml of water in a 100 ml beaker	*D* = 4.4 cm, gives *H*
Bregović^7^	30 ml of water in a beaker	*D* = 4.4 cm, gives *H*
Ghol.^22^	Sample volumes and photographs of flask	*D* = 5 cm, gives *H*
